# *CRP *polymorphisms and chronic kidney disease in the third national health and nutrition examination survey

**DOI:** 10.1186/1471-2350-12-65

**Published:** 2011-05-11

**Authors:** Adriana M Hung, T Alp Ikizler, Marie R Griffin, Kimberly Glenn, Robert A Greevy, Carlos G Grijalva, Edward D Siew, Dana C Crawford

**Affiliations:** 1 Veterans Administration Tennessee Valley Healthcare System Nashville, TN, USA; 2 Division of Nephrology, Vanderbilt University, Nashville, TN, USA; 3 Center for Human Genetics Research, Nashville, TN, USA; 4 Department of Molecular Physiology and Biophysics, Vanderbilt University, Nashville, TN, USA; 5 Department of Biostatistics, Vanderbilt University, Nashville, TN, USA; 6 Division of Pharmacoepidemiology, Department of Preventive Medicine, Vanderbilt University, Nashville, TN, USA

## Abstract

**Background:**

*CRP *gene polymorphisms are associated with serum C-reactive protein concentrations and may play a role in chronic kidney disease (CKD) progression. We recently reported an association between the gene variant rs2808630 and CKD progression in African Americans with hypertensive kidney disease. This association has not been studied in other ethnic groups.

**Methods:**

We used data from 5955 participants from Phase 2 of The Third National Health and Nutrition Examination Survey (1991-1994) to study the association between *CRP *polymorphisms and CKD prevalence in a population-based sample. The primary outcome was CKD defined as estimated glomerular filtration rate (eGFR) <60 ml/min or the presence of albuminuria. Secondary outcomes were the presence of albuminuria (any degree) and continuous eGFR. Six single nucleotide polymorphisms (SNPs) from the *CRP *gene, rs2808630, rs1205, rs3093066, rs1417938, rs3093058, and rs1800947, were evaluated.

**Results:**

*CRP *rs2808630 AG compared to the referent AA genotype was associated with CKD in non-Hispanic blacks (n = 1649, 293 of whom had CKD) with an adjusted odds ratio (OR) of 3.09 (95% CI 1.65-5.8; p = 0.001). For the secondary outcomes, rs2808630 AG compared to the referent AA genotype was associated with albuminuria with an adjusted OR of 3.07 (95% CI 1.59-5.94; p = 0.002), however not with eGFR. There was no association between the SNPs and CKD, albuminuria or eGFR in non-Hispanic whites or Mexicans Americans.

**Conclusions:**

In this cross-sectional study, the 3' flanking *CRP *gene variant rs2808630 was associated with CKD, mainly through its association with albuminuria in the non-Hispanic blacks. Despite not finding an association with eGFR, our results support our previous study demonstrating an association between *CRP *gene variant rs2808630 and CKD progression in a longitudinal cohort of African American with hypertensive kidney disease.

## Background

Chronic kidney disease (CKD) represents a major public health problem worldwide. Even in its early stages, CKD is associated with poor outcomes and premature death mainly due to cardiovascular causes [1,2]. Familial aggregation of CKD and end stage renal disease (ESRD) have been reported in populations throughout the world for all types of nephropathy, underscoring the importance of genetic factors [3-7]. There also appears to be genetic predisposition towards faster progression to ESRD which is strongly associated with race. For example, African Americans with a first degree relative with ESRD have a 9-fold increase in the risk of ESRD compared to a 3-5 fold increase in whites [8,9]. Recently, genetic variations in two candidate genes both located within a 60 kb region of chromosome 22 have been identified as high risk for non-diabetic ESRD in African Americans[10]: the nonmuscle myosin heavy chain 9 (*MYH9*)[9] and the apolipoprotein L1 (*APOL1*)[10] genes. It is possible that additional genetic variants, such as those related to inflammatory pathways, may also be associated with CKD progression through common pathways and the risk of ESRD in general.

CKD is an inflammatory process, and biomarkers of inflammation increase as kidney function declines [11,12]. Previous studies have shown that inflammation-related gene polymorphisms explain a proportion of trait variability observed for these biomarkers [1,13-19]. Given this relationship, it is likely that inflammation-related gene polymorphisms are also associated with CKD progression. We have previously demonstrated an association between a single nucleotide polymorphism rs2808630 in the *CRP *gene and CKD progression in the prospective African American Study of Kidney Disease and Hypertension (AASK) study [11], a randomized control trial designed to evaluate the effect of three different antihypertensive medications in the progression of hypertensive kidney disease [20]. In addition to being associated with decreased levels in general African American populations [15,17], this SNP has been associated with decreased CRP in the AASK study [11] suggesting that the relationship between CKD and inflammation is complex and can be influenced by genetic factors.

In the current study, we used genetic data from the Third National Health and Nutrition Examination Survey (NHANES III), a large population-based survey, to further investigate our findings in non-Hispanic blacks, non-Hispanic whites and Mexican Americans. The primary goal of this study was to test the association between six *CRP *SNPs and the presence of CKD defined as either estimated glomerular filtration rate (eGFR) <60 ml/min/m^2 ^or the presence of albuminuria according to National Kidney Foundation (NKF) consensus definitions [21]. Additionally, we evaluated the association between these polymorphisms with albuminuria (any degree) and continuous eGFR.

## Methods

### Study Population

The NHANES are surveys conducted by the National Center for Health Statistics (NCHS) at the Centers for Disease Control and Prevention (CDC) to provide national estimates of the health and nutrition of the U.S. civilian, non-institutionalized population. NHANES III is a complex, multistage survey that oversampled minorities (non-Hispanic blacks and Mexican Americans), children, and the elderly. Sample weights are provided for each participant included in the survey to correct for non-response and to correct for the fact that each participant was not selected for the survey with equal probability. Participants were consented by the CDC at the time of the survey and sample collection, and consent included the storage of data and biological specimens for future research [22].

DNA samples were generated using cell lines created from blood samples of participants older than 12 years of age during NHANES III phase 2 (1991-1994). The total number of participants in NHANES III phase 2 was 16,530 and DNA was available for 7,159 participants. To account for this difference in DNA availability, the sample weights were recalculated using previously described methods [15]. All participants with DNA available had completed a household interview and a physical examination in the Mobile Examination Center.

We excluded participants younger than 18 years of age (n = 979) as the modification of diet in renal disease (MDRD) equation is not applicable to them, 35 participants with missing serum creatinine concentrations, 71 participants with missing albuminuria measurements and normal kidney function, and 119 pregnant women. Our exclusion criteria were similar to other studies in general [23,24]. However we allowed participants age 18-20 years of age in the cohort, (different from other studies in the NHANES population [23,24]) as they are usually included in studies of CKD progression [25,26] and the MDRD equation applies to them. After exclusion criteria were applied, our final analysis included data from 5955 participants. The study protocol was reviewed and approved by the Center of Disease Control (CDC) Ethical Review Board. Because the study investigators did not have access to personal identifiers, the Vanderbilt University Internal Review Board and the VA Tennessee Valley Healthcare System approved the protocol as non-human subject research under the exempt category.

#### Measurements and Definitions

***The primary outcome ***for this study was CKD defined as either an eGFR <60 ml/min or the presence of albuminuria in accordance to the NKF consensus definition which classifies all individuals with evidence of kidney damage (e.g. albuminuria) as having chronic kidney disease, irrespective of the level of GFR [21]. The focus of our study was to identify SNPs associated with common pathways for advanced CKD, and albuminuria is one of the main risk factors for CKD progression [2,27]. ***Secondary outcomes ***were a) albuminuria defined using sex-specific cut-offs of urine albumin creatinine ratio (ACR) ≥ 17 mg/g for men and ≥ 25 mg/g for women [24,28,29] and b) continuous eGFR. The choice of analyzing eGFR as a continuous trait was made to maximize the information available in the dataset, as there was a small number of participants with low GFR. We also performed an sensitivity analysis where eGFR was dichotomized as < or ≥ 60 ml/min.

NHANES creatinine was calibrated to the creatinine values of the Cleveland Clinic Laboratory [creatinine calibration formula: MDRDcr = -0.184 + 0.96 *NHANES III uncalibrated creatinine]. eGFR was calculated using the MDRD four-component equation: eGFR (mL/min/1.73 m2) = 175 × (serum creatinine)^-1.154 ^× (Age)^-0.203 ^× (0.742 if female) × (1.210 if African American). Calibration formula: MDRDcr = -0.184 + 0.96 *NHANES III uncalibrated creatinine. Urine albumin and creatinine were measured from a single random morning voided urine sample. Samples were obtained by a clean catch technique and shipped overnight on dry ice to a central laboratory and frozen for a later use. Urine albumin concentration was measured by means of solid-phase fluorescent immunoassay. Urine creatinine concentration was measured using the Jaffé method. Urine albumin creatinine ratio (ACR) is expressed in units of milligrams per gram. Similarly, CKD stages were categorized based on the current NKF classification system.

### Genotype data

As previously reported, the *CRP *gene (2.3 kb) is located on chromosome 1q21-1q23. We accessed the genotype data for six *CRP *SNPs (rs1417938, rs1205, rs1800947 (Leu184Leu), rs2808630, rs3093058, and rs3093066) through the NHANES Genetic database maintained by the National Center for Health Statistics at the Centers for Disease Control and Prevention. All six SNPs selected were previously genotyped by Crawford et al [15] using Applied Biosystems TaqMan and Epoch MGB Eclipse Probe Systems (rs1800947). As previously reported [15], the overall average SNP genotyping call rate was 95%, and no SNP was out of Hardy Weinberg Equilibrium at p < 0.001 in all three subpopulations tested

### Covariate Definitions

We ascertained known risk factors for CKD using NHANES measurements. Race/ethnicity was self-assigned as non-Hispanic white, non-Hispanic black, Mexican American, or other. C-reactive protein (CRP) assay used in for NHANES was a low sensitivity assay, with a lower limit of detection of 0.22 mg/dL. CRP values were right skew and were log transformed for the analysis. Diabetes mellitus (DM) was defined as a self-reported physician diagnosis of diabetes, use of diabetes medications (insulin or pills for diabetes), a plasma glucose from-first venipuncture >126 mg/dl or a random glucose from the biochemistry profile > 200 mg/dl. Hypertension was defined as any use of antihypertensive medications, the average of two systolic blood pressure measurements of 140 mm Hg or greater, or diastolic blood pressure of 90 mm Hg or greater taken during the examination. Smoking status was defined as "current" if the participant answered "yes" to the question "do you smoke now" or if the participant had cotinine levels >15 ng/mL. Body mass index (BMI) was classified as obese (≥30 kg/m^2^) or not obese. Triglycerides, calculated LDL cholesterol, and HDL cholesterol were treated as continuous variables. Cardiovascular disease (CVD) was considered positive if the participant reported ever having been told that he or she had had a heart attack, heart failure, or stroke or had undergone a surgical or percutaneous procedure for any of these conditions.

### Statistical analysis

All results were weighted to account for the sampling design of NHANES III, which were recalculated by the CDC for NHANES III genetic study. All analyses were stratified by self reported race/ethnicity. Unadjusted and adjusted logistic regressions were conducted assuming an additive genetic model for each SNP (that is, with a change in the odds ratio or beta-coefficient per copy of the minor allele). Our prior research indicated that minor allele "G" is the "at-risk" allele for CKD progression compared with the major allele "A". Hence, SNPs were treated as categorical variables and coded as follows: 0 = homozygous major, 1 = heterozygous, and 2 = homozygous minor; the homozygous major category was considered the reference level. The primary outcome was CKD (any stage of CKD) treated as a binary outcome given the small number of participants with advance stages of CKD 4-5. For the secondary outcomes, continuous GFR was log transformed. Albuminuria was defined as a binary outcome (any degree of albuminuria). All analyses were performed either remotely using the Analytic Data Research by Email (ANDRE) portal of the CDC Research Data Center (RDC) in Hyattsville, MD or in person at the RDC using STATA 10 MP(College Station, TX), SAS/Genetics 9.1.3 (SAS Institute, Cary, NC), and SUDAAN 10 (Research Triangle Institute, NC). Our power calculation was focused in the association of the rs2808630 polymorphims and CKD in African Americans, based in our previous study (12) with a minor allele frequency of 0.14 for NHANES III. We estimated that we needed 253 African Americans with CKD and 253 African Americans without CKD in order to detect a genotype risk of 1.6 or higher, using an additive model. This model was chosen base in our previous study (12). Additionally given our small sample of African Americans with CKD (n = 293) we estimated that we will be under power for a dominant model requiring at least 354 controls and cases for the same genotype risk. We considered significance for a two sided alpha of 0.05 for all analyses and Bonferroni-adjusted alpha of 0.002 (0.05/29).

## Results

Participants' characteristics are shown in table 1. From the 5955 participants that met criteria for our study, a total of 1,175 (20%) qualified as having CKD. More specifically, 7% of the participants (n = 406) had a GFR < 60 ml/min and 16% (n = 961) had albuminuria. Chronic kidney disease patients were older, 81% (n = 951) had albuminuria, had a higher prevalence of diabetes mellitus, hypertension, cardiovascular disease, higher body mass index, higher CRP levels, and lower smoking exposure compared to participants without CKD. Genotype frequencies by race/ethnicity are displayed in table 2. There were no statistically significant differences in the clinical characteristics among the different race/ethnicity groups by CKD status (additional file 1, table S1). Genotype frequencies by race for participants with and without CKD are displayed in table 2. Genotype frequencies by race for participants with and without albuminuria are displayed in additional file 1 table S4.

**Table 1:** Characteristics of the study participants ≥ 18 years of age, NHANES III DNA bank, 1991-1994, stratified by chronic kidney disease (CKD) status.

Characteristics	Total (n = 5955)	§ CKD (n = 1175)	No CKD (n = 4780)	p-value
Age, median (IQR) years	42 (30, 62)	67 (48,77)	38 (28,48)	<0.0001
Gender (males) (%)	44	42	44	0.01
Race/Ethnicity (%)				0.02
Non-Hispanic White % (n)	39% (2338)	47% (556)	37% (1782)	
Non-Hispanic Black % (n)	28% (1649)	25% (293)	28% (1356)	
Mexican American % (n)	28% (1685)	23% (272)	30% (1413)	
Others % (n)	5% (283)	5% (54)	5% (229)	
Serum Creatinine, mg/dL, median (IQR)	1 (0.9, 1.2)	1.2 (1,1.4)	1 (0.9,1.2)	<0.0001
Median eGFR ml/min/1.73 m2 (IQR)	93 (78, 109)	66 (53,87)	88 (77,104)	<0.0001
Urine albumin to creatinine ratio (IQR)	5.38 (2.66, 11.79)	35 (17, 87)	4 (21,74)	<0.0001
Albuminuria				<0.0001
None, % (n)	84% (5004)	19% (224)	100% (4780)	
Microalbuminuria, % (n)	14% (813)	69%(813)	0 (0)	
Macroalbuminuria, % (n)	2% (138)	12%(138)	0 (0)	
Any form of albuminuria, % (n)	16% (951)	81%(951)	0 (0)	
Low GFR				
GFR < 60 ml/min (%)	7	35	0	<0.0001
CKD stage III (eGFR 30-59 ml/min) (%)	6.5	3	0	
CKD stage IV (eGFR 15-29 ml/min) (%)	0.4	1	0	
CKD stage V (eGFR < 15 ml/min) (%)	0.1	1	0	
Systolic BP median (IQR), mmHg	119 (108, 134)	136 (120, 155)	117 (108,129)	<0.0001
Diastolic BP median (IQR), mmHg	62 (64, 80)	74 (66, 83)	72 (65,79)	0.07
Hypertension, %	22	46	15	<0.0001
BMI, median (IQR)	26.5 (23, 30)	27 (24, 31)	26 (23, 30)	0.02
C-reactive protein level (%)				<0.0001
<0.22 mg/dl (%)	62	53	65	
0.22-0.99 mg/dl (%)	29	34	27	
>1.00 mg/dl (%)	9	13	7	
Current Smoking (%)	20	18	19	0.002
Prevalent CHD, (%)	8	19	4	<0.0001
LDL median (IQR),, mg/dL	122 (99, 146)	133 (106, 157)	120 (98, 144)	0.13
Diabetes (%)	11	26	7	<0.0001

*Categorical variables were tested using the Cochran-Mantel-Haenszel Test and continuous variables were tested using the Taylor Linearization method with SUDAAN. All tests were weighed.

§ CKD defined as either by decrease glomerular filtration rate (eGFR) or by the presence of albuminuria

**Table 2:** *CRP *polymorphisms, location and genotype frequencies for all participants by CKD status.

*SNP*	rs2808630			rs1205			rs1800947*			rs3093058			rs3093066			rs1417938		
*Location*	3' flanking region			3' flanking region			Exon 2			5' flanking region			5' flanking region			Intron 1		
*Genotypes*	AA	AG	GG	GG	AG	AA	GG	CG	CC	AA	AT	TT	CC	AC	AA	AA	AT	TT
***Non-Hispanic Whites***																		
*N*	270	221	43	241	236	57	475	**	**	537	5	-	498	9	-	259	239	48
*CKD, GFP*	50.75	42.68	6.57	46.17	42.19	11.63	93.19	**	**	98.36	1.64	-	97.69	2.31	-	47.44	43.7	8.79
*N*	867	686	133	694	808	190	1544	**	**	1718	6	-	1610	15	-	851	730	148
*No-CKD,GFP*	52.13	40.38	7.49	42.16	46.51	11.33	94.43	**	**	99.55	0.45	-	98.94	1.06	-	49.22	42.22	8.56
***Non-Hispanic Blacks***																		
	**rs2808630**			**rs1205**			rs1800947*			**rs3093058**			**rs3093066**			**rs1417938**		
*N*	181	96	8	190	86	9	**	**	-	212	71	8	154	105	15	221	62	**
*CKD, GFP*	61.45	36.4	2.14	67.06	29.39	3.55	99	**	-	76.01	22.89	1.1	56.35	38.37	5.28	77.75	20.65	**
*N*	950	323	26	804	397	79	**	**	-	915	356	34	751	443	67	1024	272	21
*No-CKD,GFP*	72.24	25.42	2.34	62.68	31.26	6.06	99	**	-	69.67	27.66	2.67	60.67	34.12	5.21	77.54	21.75	0.4
***Mexicans Americans***																		
	**rs2808630**			**rs1205**			rs1800947*			**rs3093058**			**rs3093066**			**rs1417938**		
*N*	167	89	11	102	137	31	253	7	-	260	8	**	269	**	**	110	130	24
*CKD, GFP*	61.12	33.75	5.13	42.21	47.09	10.70	97.66	2.3	-	95.81	3.91	**	99.25	**	**	41.67	49.23	9.1
*N*	852	434	57	562	621	168	1266	54	-	1348	37	**	1308	**	**	591	616	155
*No-CKD,GFP*	62.48	33.04	4.48	41.65	45.65	12.7	95.6	4.4	-	97.08	2.92	**	96.59	**	**	43.39	45.23	11.3

** Data user agreement with the CDC does not allow outputs with counts less than 5.

***N: ***number of individuals with the specific genotype. ***GFP: ***genotype frequency percent

### *CRP *genotypes and CKD defined as either by the presence of albuminuria or low GFR

Among non-Hispanic blacks, rs2808630 was associated with CKD both in the unadjusted (table 3) and adjusted analysis (table 4). More specifically, the AG genotype had an unadjusted odds ratio of 1.68 (95% CI: 1.34-2.12; p = 0.0001) for the presence of CKD compared to the AA genotype (table 3). Similarly the AG genotype was associated with CKD with an odds ratio of 2.94 (95% CI: 1.61 to 5.38; p = 0.001) compared to the AA genotype for the fully adjusted model, for other known risk factors for CKD. Both of these associations remained significant even after Bonferroni adjustment for the significance level (p ≤ 0.002). This significant association did not change when adjusting for CRP (table 4). Other independent predictors in the model for the SNP rs2808630 and CKD included age 1.05 (1.03,1.07), hypertension 2.21 (1.33, 3.08) and diabetes 2.44 (1.11, 5.35). History of cardiovascular disease 1.42 (0.66,3.0.8), obesity 0.77 (0.48,1.25), LDL cholesterol mg/dL 1.0 (0.9, 1.01), smoking 1.11 (0.63, 1.97) and CRP levels (mg/dL) 0.96 (0.81, 1.14) were not independent predictor in this model.

**Table 3:** Unadjusted and weighed association of *CRP *gene polymorphism and CKD, additive genetic model

Presence of CKD (logistic regression) for each SNP									
	**No**			**No**			**No**		
		**Non-Hispanic Whites**	¥ **p value**		**Non-Hispanic Blacks**	¥ **p value**		**Mexican American**	¥ **p value**

***rs2808630***									
AA	*1137*	*ref*		*1131*	*ref*		*1019*	*Ref*	
AG	*907*	1.09 (0.78-1.52)	0.6	*419*	1.68 (1.34 - 2.12)	0.0001*	*523*	1.04(0.58-1.88)	0.9
GG	*176*	0.90 (0.52-1.57)	0.7	*34*	1.08 (0.43 - 2.71)	0.9	*68*	1.17(0.57-2.40)	0.7
***rs1205***									
GG	*935*	*ref*		*994*	*ref*		*664*	*Ref*	
AG	*1044*	0.88 (0.61-1.28)	0.5	*483*	1.60 (0.66-3.88)	0.3	*758*	1.22 (0.76-1.97)	0.4
AA	*247*	1.07 (0.77-1.48)	0.7	*88*	1.82 (0.82-4.06)	0.1	*199*	1.20(0.77-1.89)	0.4
***rs1417938***									
AA	*1110*	*ref*		*1245*	*ref*		*701*	*Ref*	
AT	*969*	1.07 (0.76-1.50)	0.7	*334*	1.08(0.080-1.44)	0.6	*746*	1.21 (0.92-1.58)	0.2
TT	*196*	1.08 (0.61-1.93)	0.8	*23*	0.43(0.09-1.99)	0.3	*179*	1.10 (0.48-2.50)	0.8
***rs1800947***									
GG	*2019*	*ref*		*1508*	*ref*		*1519*	*Ref*	
CG	**	1.21 (0.56 - 2.66)	0.6	*20*	0.55 (0.13-2.26)	0.4	*61*	0.52 (0.20-1.38)	0.2
***rs3093058***									
AA	*2255*	*ref*		*1127*	*ref*		*1608*	*Ref*	
AT	*11*	3.69 (0.74-18.44)	0.09	*427*	0.76 (0.60-0.96)	0.02	**	1.36 (0.59-3.15)	0.5
TT	-	_		*42*	0.38 (0.13-1.07)	0.07	**	_	
***rs3093066***									
CC	*2108*	*ref*		*905*	*ref*		*1577*	Ref	
CA	*24*	2.20 (0.65-7.47)	0.2	*548*	1.11 (0.56-2.21)	0.8	**	0.22 (0.05-0.97)	0.03
AA	-	***_***		*82*	0.92 (0.54-1.55)	0.7	**	_	

¥ Unadjusted for multiple comparisons

*Statistical significance with Bonferroni correction (α/n = 0.05/29) p-value ≤ 0.002

**Table 4:** Adjusted and weighted association between *CRP *polymorphism rs2808630 and rs3093058 and CKD in African Americans Participants.

*Models*	*Genotype*	*OR*	*95% CI*	¥ *p-value*
**rs2808630**				

*Fully adjusted model*	AA	ref**		
	AG	2.94	1.61, 5.38	0.001*
	GG	2.22	0.68, 7.23	0.2

*Fully adjusted model plus Log transformed CRP*	AA	ref**		
	AG	3.09	1.65, 5.80	0.001*
	GG	2.41	0.79, 7.39	0.1

***rs3093058***				

*Fully adjusted model*	AA	ref**		
	AT	0.44	0.49,1.44	0.5
	TT	0.15	0.02, 1.36	0.09
*Fully adjusted model plus Log transformed CRP*	AA	ref**		
	AT	0.8	0.45, 1.41	0.4
	TT	0.15	0.02,1.39	0.09

¥ Unadjusted for multiple comparisons

*Statistical significance with Bonferroni correction (α/n = 0.05/29) p-value ≤ 0.002

Number of participants by rs2808630 genotype: AA = 1131; AG 419, and GG = 34

**ref. reference group homozygous for the major allele.

Fully adjusted model: adjusted for age, sex, diabetes, hypertension, cardiovascular disease, BMI, smoking and LDL cholesterol

In contrast, *rs3093058 *was associated with a decreased risk of CKD in the unadjusted analysis [AG vs. AA genotype, 0.76 (95% CI 0.60-0.96; p = 0.02)] (table 3). However, this association was not significant after Bonferroni adjustment or after adjustment for known risk factors for CKD. None of the other SNPs were associated with the risk of CKD in African Americans. There was no association observed between the studied SNPs and CKD in non-Hispanic whites or Mexicans Americans.

### CRP genotypes and continuous GFR by MDRD equation

We examined the association between the selected *CRP *SNPs and continuous eGFR. There was no association detected with continuous eGFR and the tested *CRP *SNPs in the unadjusted or fully adjusted model (table 5). Additional unadjusted logistic regression for low eGFR (GFR < 60 ml/min) was performed as an alternative analysis for all *CRP *SNPs with no association detected **(data not shown)**.

**Table 5:** Association between CRP variants and eGFR-MDRD

	linear regression to eGFR (log transformed) for each SNP					
	**Non-Hispanic Whites**		**Non-Hispanic Blacks**		**Mexican Americans**	
	**β(95% CI)**	¥ **p-value**	**β (95% CI)**	¥ **p-value**	**β(95% CI)**	¥ **p-value**
	
**rs2808630**						
Unadjusted	-0.001 (-0.02, 0.01)	0.55	-0.01 (-0.04,0.02)	0.61	-0.01 (-0.01, 0.03)	0.54
Fully adjusted	-0.01 (-0.04, 0.02)	0.46	0.00 (-0.04, 0.04)	0.94	0.01 (-0.01, 0.04)	0.20
**rs1205**						
Unadjusted	-0.001 (-0.02, 0.01)	0.76	-0.01 (-0.03, 0.02)	0.53	-0.001(-0.01, 0.02)	0.55
Fully adjusted	-0.001 (-0.02, 0.02)	0.84	-0.01 (-0.04, 0.01)	0.32	0.01 (-0.02, 0.04)	0.55
**rs1417938**						
Unadjusted	0.001 (-0.02, 0.02)	0.72	-0.01(-0.04, 0.02)	0.54	-0.01 (-0.03, 0.01)	0.51
Fully adjusted	0.01 (-0.01, 0.03)	0.35	-0.02 (-0.05, 0.01)	0.18	-0.001(-0.03, 0.03)	0.84
**rs1800947**						
Unadjusted	-0.001 (-0.05, 0.04)	0.87	0.01 (-0.13, 0.16)	0.84	0.03 (- 0.02, 0.07)	0.26
Fully adjusted	0.01(-0.07, 0.09)	0.75	0.04 (-0.20, 0.27)	0.75	0.01 (-0.04, 0.06)	0.72
**rs3093058**						
Unadjusted	-0.08 (-0.31, 0.14)	0.45	0.01 (-0.01, 0.04)	0.36	0.01 (-0.05, 0.06)	0.78
Fully adjusted	-0.04 (-0.13, 0.05)	0.36	-0.01 (-0.04, 0.02)	0.63	-0.03 (-0.11, 0.04)	0.34
**rs3093066**						
Unadjusted	0.02 (-0.19, 0.23)	0.86	0.02 (-0.01, 0.04)	0.16	-0.03 (-0.09, 0.03)	0.37
Fully adjusted	0.06 (-0.13, 0.25)	0.53	0.01 (-0.02, 0.03)	0.68	-0.01 (-0.11, 0.08)	0.78

¥ Unadjusted for multiple comparisons

Fully adjusted: age, gender, diabetes, hypertension, cardiovascular disease, BMI, smoking, LDL cholesterol, log transformed C reactive protein and albuminuria

### *CRP *genotypes and albuminuria

We further evaluated the association between the *CRP *SNPs with albuminuria (any degree of albuminuria versus none). The rs2808630 SNP was the only *CRP *SNP significantly associated with albuminuria in non-Hispanic blacks. To examine the association of the rs2808630 with albuminuria, we adjusted all our models for eGFR. In the minimally adjusted analysis (model 1), the AG genotype had an OR 1.68 (95% CI 1.27-2.23; p = 0.0009) for the presence of albuminuria compared to the AA genotype. Similarly, the AG genotype of rs2808630 was associated with albuminuria in the fully adjusted model with an OR of 3.07 (95%CI 1.59-5.94; p = 0.002) compared to the AA genotype (table 6). These associations were significant after Bonferroni adjustment (p ≤ 0.002). Other independent predictors in the model for the SNP rs2808630 and albuminuria included age (years) 1.03 (1.01,1.06), hypertension 2.42 (1.35, 4.35) and diabetes 2.52 (1.12, 5.67). History of cardiovascular disease 1.47 (0.67, 3.26), body mass index(BMI) greater than thirty 0.82 (0.46, 1.41), LDL cholesterol mg/dL 1.0 (0.99, 1.00), smoking 1.18 (0.67, 2.06) and CRP levels (mg/dL), 1.24 (0.90, 1.70) were not independent predictor in this model. There was no association observed between the studied SNPs and albuminuria in non-Hispanic whites or Mexicans Americans.

**Table 6:** Adjusted and weighted association between *CRP *polymorphism rs2808630 and albuminuria in African Americans Participants.

*Models*	*Genotype*	*OR*	*95% CI*	¥ *p-value*
***rs2808630***				

*Adjusted for Age and gender (Model 1).*	AA	ref*		
	AG	1.68	1.27, 2.23	0.0009
	GG	0.99	0.47, 2.28	0.9

*Adjusted for risk factors*	AA	ref*		
	AG	2.95	1.54, 5.67	0.002
	GG	1.78	0.50, 76.34	0.4

*Adjusted for risk factors and Log transformed CRP (Model 2).*	AA	ref*		
	AG	3.07	1.59, 5.94	0.002
	GG	1.91	1.00, 6.54	0.3

*ref. reference group homozygous for the major allele.

¥ Unadjusted for multiple comparisons

* Statistical significance with Bonferroni correction (α/n = 0.05/29) p-value ≤ 0.002

Odd ratios are adjusted for:

***Model 1: ***adjusted for age, sex and GFR.

***Model 2: ***age, gender, diabetes, hypertension, cardiovascular disease, BMI, cotinine levels, NSAIDS exposure, LDL cholesterol, GFR and log transformed C reactive protein.

There were no statistically significant differences in the demographics or clinical characteristics among the different rs2808630 genotypes (table 7).

**Table 7:** Clinical Characteristics of African American Participants of the NHANES III Genetic Study (1991-1994) by rs2808630 genotype.

	AA (n = 1131)	AG (n = 416)	GG (n = 34)	p-value
Age, years, median (IQR)	33 (20,46)	34 (22,47)	38(29,55)	0.2
Gender, male (%)	43	42	50	0.9
Hypertension (%)	23	24	29	0.3
Diabetes (%)	9	11	* (*)	0.6
Cardiovascular Disease (%)	6	5	* (*)	0.8
Serum Creatinine, mg/dL (calibrated)	1.1 (0.9,1.2)	1 (0.9,1.2)	1.1 (1, 1.2)	0.39
Urine albumin to creatinine ratio	4.7 (2.2, 104)	5.2 (2.3, 15)	6.9 (3.4, 25)	0.03
Body Mass Index Kg/m2	26 (22, 30)	26 (22, 32)	26 (22, 32)	0.06
LDL cholesterol, mg/dL	121 (98, 145)	115 (95, 142)	100 (80, 169)	0.9
Smoking (%)	37	38	38	0.8

Categorical variables were tested using the Cochran-Mantel-Haenszel Test and continuous variables were Tested using the Taylor linearization method in SUDAAN with all analysis weighed.

## Discussion

In this nationally-representative population, we show an association between the *CRP *SNP rs2808630 and the presence of CKD in non-Hispanic black participants defined either by low GFR < 60 ml/min or the presence of albuminuria. This result is concordant with the findings of our previous study in which rs2808630 was associated with CKD progression in a longitudinal prospective CKD cohort from the African American Study of Kidney Disease and Hypertension[11].

CKD is multifactorial, occurring in the context of chronic co-morbid conditions such as diabetes mellitus, cardiovascular disease, obesity and hypertension. Since all of these conditions impact vascular inflammation, CKD is considered as an inflammatory condition [12,30]. Based on these observations, one can speculate that individuals prone to a higher inflammatory response due to genetic predisposition would have a higher risk of CKD progression. However, our current and previous study showed that the minor allele of SNP rs2808630, which is associated with lower CRP levels in the general population (18, 26, 20, 21), is associated with CKD, now in two independent cohorts[11] along with another replication study [31]. The association between rs2808630 and CKD status remained significant after adjustment for CRP levels, suggesting that the pathway for this association is not mediated by CRP. On the other hand, it may be related to other immune-modulatory molecules of the cytokine network, which involves a highly complex interaction of multiple cascades of gene activation and suppression [32].

Another possible explanation for our observations is that the association between inflammation and CKD is the consequence of the multiple co-morbidities and decreased clearance from the kidney. This might suggest that the inflammatory response itself may not be related to a genetic predisposition towards disease progression in kidney disease. It is also possible that the rs2808630 is in linkage disequilibrium with another SNP that increases the risk of CKD. *CRP *rs2808630 is located in the 3' flanking region of the gene and is not in strong linkage disequilibrium with any common variation found within 50 kb in the Yoruba International HapMap dataset, although it is in moderate LD (r^2^~0.5) with three intergenic common SNPs [33]. We cannot rule out the possibility that other common SNP(s) outside the 50 kb boundary may be in LD with rs2808630 or that a less common SNP (in the 1%-5% range) is in LD with rs2808630. After a preliminary exploration of the newly released 1000 Genomes Project Data for Yoruba [34] the latter possibility seems unlikely (data not shown).

In order to better understand the association of *CRP *rs2808630 with CKD, we studied the association of the rs2808630 with albuminuria and with continuous eGFR as independent outcomes. *CRP *rs2808630 was also associated with albuminuria. Albuminuria is a strong risk factor for CKD progression and could potentially represent a pathway for the association with CKD progression. Although genetic basis for GFR traits [35](9) and albuminuria traits [36,37] have been recently identified and may differ, it has been recently highlighted that information regarding albuminuria should be incorporated in the CKD staging system in order to be able to predict those at risk for progression [27]. This relevant clinical observation highlights that gene variants associated with albuminuria may be part of the common pathway for CKD progression and underscores the need to include albuminuria in the outcome when studying genetic susceptibility for CKD progression through common pathways.

Finally, individuals with this *CRP *genotype may respond less to other interventions compared with patients that have the referent genotypes at these SNPs. Our previous study findings in the AASK trial suggested that African Americans with the minor allele for the rs2808630 benefited less from ACE inhibition. Unfortunately, NHANES III lacks detailed medication data, and we were not able to test for this association.

Recently, genome-wide association studies (GWAS) studies have identified a number of potential candidate genes and genomic regions that may contribute to the pathogenesis of CKD(9, 10, 12). Two important susceptibility genes for CKD progression to ESRD have been identified in African Americans for "non-diabetic kidney disease"[10]. These include *MYH9 *[9] and *APOL1 *[10]. In addition to the genes implicated in CKD progression to ESRD, GWAS meta-analyses has also yielded a large number of potential gene variants that are associated with both creatinine-based and cystatin C-based eGFR[35]. However, taken all together, these SNPs explain only about 1.43% of the GFR variability at the population level, suggesting that many other genetic components of kidney function remain to be identified. Furthermore it has been hypothesized that there are genes involved in disease initiation only (i.e *UMOD*) [35] and other genes involved in progression to ESRD.

The main strength of this study is that it is performed in a large population-based, nationally representative and ethnically diverse sample, which is rich in phenotypic information, allowing us to adjust in our analysis for the main risk factors known to be associated with CKD progression. However, our study is not without limitations. NHANES III is a population based sample designed to represent the health status of the non-institutionalized US population which follows a cross-sectional design and precludes the possibility of studying gene effect on disease progression. Secondly, eGFR was calculated using a single creatinine measurement. Given some variability involved in the measurement, a single value may increase the risk of misclassification of participants without CKD as CKD and overall dilute the association. Thirdly, because this is a population-based sample, the majority of the participants had normal kidney function (mean GFR 95 ± 25 ml/min) and only 7% had low eGFR, with the vast majority of them classified as stage 3. This may have limited our ability to detect an association with eGFR. In contrast, our previous study [11] was performed in a population of only African Americans, all of whom had established CKD (mean baseline GFR 43 47 ± 13.5 ml/min) followed logintudinally and with repeated measurements of the outcome using the gold standard to measure GFR (iothalamate I-125). Overall, these two studies are complementary to each other (a comparison of the difference between populations studies, design and outcome ascertaiment between AASK and NHANES is provided in our additional file1 tables S2 and S3). The fact that we were able to demonstrate an association with albuminuria is also highly relevant as this may potentially explain the relationship with CKD progression via a common pathway.

## Conclusion

The *CRP *gene variant rs2808630 was associated with the presence of CKD in non-Hispanic black participants. Larger longitudinal studies of individuals with established CKD are needed to further evaluate the role of *CRP *polymorphisms in CKD progression in an ethnically diverse population, including non-Hispanic whites and Mexican Americans. Further studies are also required to evaluate if this association is with CKD in general or with specific disease etiologies such as diabetes and hypertension. Finally, pharmacogenetic studies evaluating the role of *CRP *polymorphisms in the response to specific medications such as ACE inhibition with regards to renoprotection are also needed.

## Competing interests

The authors declare that they have no competing interests.

## Authors' contributions

AMH formulated the hypothesis, led the development of the statistical plan and wrote part of the manuscript. TAI, gave advice in the interpretation of the results and in the manuscript writing. MRG gave advice the statistical plan and help in the interpretation of the results. KG performed the statistical analysis. RG helped in the analytical dataset creation & gave advice the statistical plan. CG helped with the dataset creation and help in the interpretation of results. ES help with results interpretation and actively participated in writing part of the manuscript. DCC gave advice in the statistical plan and help in the interpretation. All authors critically reviewed and approved the manuscript

## Pre-publication history

The pre-publication history for this paper can be accessed here:

http://www.biomedcentral.com/1471-2350/12/65/prepub

## Supplementary Material

Additional file 1 **Table S1; Clinical characteristics by CKD status and by race/ethnicity**. This table compares the clinical characteristics of individuals with and without CKD by race group. Table S2: Comparison in the study design, population and outcome ascertainment between NHANES III DNA bank (1991-1994) and the African American Study in Kidney Disease and Hypertension. This table provides a comparison of NHANES III DNA subgroup and the AASK trial. Table S3: Clinical characteristics of the AASK participants by rs2808630 genotypes This table provides the clinical characteristics by rs2808630 by genotype for the AASK participants Table S4: *CRP *polymorphisms, genotypes and weighed frequencies for all participants with and without albuminuria Genotype frequencies by race and by albuminuria status for all genotype SNPs. Click here for file
