# Supplementary material for: CRP polymorphisms and chronic kidney disease in the third national health and nutrition examination survey
Source: BMC Med Genet. 2011 May 11;12:65. doi: 10.1186/1471-2350-12-65 (PMC3119179; doi:10.1186/1471-2350-12-65)
Supplement: Additional file 1 — Table S1; Clinical characteristics by CKD status and by race/ethnicity. This table compares the clinical characteristics of individuals with and without CKD by race group. Table S2: Comparison in the study design, population and outcome ascertainment between NHANES III DNA bank (1991-1994) and the African American Study in Kidney Disease and Hypertension. This table provides a comparison of NHANES III DNA subgroup and the AASK trial. Table S3: Clinical characteristics of the AASK participants by rs2808630 genotypes This table provides the clinical characteristics by rs2808630 by genotype for the AASK participants Table S4: CRP polymorphisms, genotypes and weighed frequencies for all participants with and without albuminuria Genotype frequencies by race and by albuminuria status for all genotype SNPs. [file 1471-2350-12-65-S1.DOC]

Supplemental tables

Supplemental table 1

Clinical characteristics by CKD status and by race/ethnicity.

|  | Non-Hispanic Whites | | Non-Hispanic Blacks | | Mexican Americans | |
| --- | --- | --- | --- | --- | --- | --- |
|  | No-CKD  1782 | CKD  556 | No-CKD  1356 | CKD  293 | No-CKD  1413 | CKD  272 |
| Age | 49±19 | 69±16 | 38±15 | 54±18 | 39±16 | 55±19 |
| Gender, % male | 41 | 38 | 42 | 48 | 52 | 48 |
| Systolic BP, mmhg | 121±19 | 139±24 | 120±18 | 141±28 | 118±17 | 139±28 |
| Diastolic BP, mmhg | 72±11 | 72±14 | 73±13 | 78±17 | 71±11 | 75±15 |
| Hypertension (%) | 18 | 46 | 18 | 48 | 12 | 48 |
| Diabetes (%) | 6 | 20 | 6 | 24 | 8 | 37 |
| Cardiovascular  Disease (%) | 6 | 23 | 4 | 15 | 4 | 16 |
| Serum Creatinine, mg/dL | 1.04±0.15 | 1.28±0.5 | 1.1±0.19 | 1.4± 0.9 | 0.99±0.2 | 1.13±0.5 |
| Urine albumin to creatinine ratio, mg/g | 6±4.6 | 106±367 | 5±4.7 | 197±418 | 5.4±4.7 | 180±362 |
| Body mass index  (kg/m^2^) | 27±5 | 27±6 | 28±6 | 29±7 | 27±5 | 29±6 |
| LDL  Cholesterol, mg/dL | 128±36 | 135±41 | 121±38 | 132±45 | 119±33 | 128±37 |

Supplemental table 2

Comparison in the study design, population and outcome ascertainment between NHANES III DNA bank (1991-1994) and the African American Study in Kidney Disease and Hypertension.

|  | NHANES III  DNA bank sample  (1991-1994) | AASK Study |
| --- | --- | --- |
| Design | Cross-sectional | Longitudinal |
| Population | National representative sample of the US non-institutionalized  population | African Americans with established Hypertensive  Chronic Kidney Disease. |
| Measurements | 1) Serum creatinine, measured at one single point in time. Creatinine based GFR estimate using the MDRD formula  2) Urine to albumin to creatinine ratio, one single urine sample | 1)GFR measured by  Iothalamate-I-125 clearance  2)Urine protein at baseline estimated from the average of two 24 hour urine collection |
| Outcome | 1) Serum creatinine, measured at one single point in time. Creatinine based GFR estimate using the MDRD formula  2) Urine to albumin to creatinine ratio, one single urine sample | Time to event analysis of halving your GFR, end stage renal disease or death |

Supplemental table 3.

Clinical characteristics of the AASK participants by rs2808630 genotypes

|  | *rs2808630 genotypes* | | | p-value |
| --- | --- | --- | --- | --- |
|  | GG | AG | AA |
| N | 15 | 191 | 436 |
| Age (yr) | 48± | 55± | 54± | 0.23 |
| Gender, % male | 40 | 59 | 60 | 0.3 |
| Serum creatinine (mg/dl) | 1.8 1.4 2.1 | 1.8 1.5 2.4 | 1.8 1.5 2.3 | 0.79 |
| 24 hours urine at baseline (g/d) | 0.44±0.73 | 0.54±0.53 | 0.48±0.92 | 0.4 |
| Systolic BP  At randomization,  mmgh | 149±23 | 151±25 | 150±24 | 0.5 |
| Diastolic BP  At randomization,  mmgh | 100±16 | 95±14 | 96±14 | 0.09 |
| History of  Heart disease, % | 40 | 50 | 50 | 0.74 |
| Body mass index (kg/m^2^) | 34±6 | 31±7 | 31±7 | 0.09 |
| Smoking, % | 40 | 29 | 27 | 0.58 |
| GFR slope  (ml/min/m^2^) | -3.03 IQR: -5.2, -0.22 | -2.18 IQR: -3.6, -0.26 | -1.87 IQR: -3.3, -0.26 | 0.07 |

*Supplemental table 4. CRP* polymorphisms, genotypes and weighed frequencies for all participants with and without albuminuria

| ***SNP*** | **rs2808630** | | |  | **rs1205** | | |  | **rs1800947*** | | |  | **rs3093058** | | |  | **rs3093066** | | |  | **rs1417938** | | |
| --- | --- | --- | --- | --- | --- | --- | --- | --- | --- | --- | --- | --- | --- | --- | --- | --- | --- | --- | --- | --- | --- | --- | --- |
| ***Location*** | 3’ flanking region | | |  | 3’ flanking  region | | |  | Exon 2 | | |  | 5’ flanking  region | | |  | 5’ flanking  region | | |  | Intron 1 | | |
| ***Genotypes*** | **AA** | **AG** | **GG** |  | **GG** | **AG** | **AA** |  | **GG** | **CG** | **CC** |  | **AA** | **AT** | **TT** |  | **CC** | **AC** | **AA** |  | **AA** | **AT** | **TT** |
| **Genotype frequencies % (GFP)** | | | | | | | | | | | | | | | | | | | | | | | |
| ***Non-Hispanic Whites*** | | | | | | | | | | | | | | | | | | | | | | | |
|  | **rs2808630** | | |  | **rs1205** | | |  | **rs1800947*** | | |  | **rs3093058** | | |  | **rs3093066** | | |  | **rs1417938** | | |
| *N* | 189 | 157 | 27 |  | 167 | 163 | 35 |  | 324 | ** | ** |  | 370 | ** | - |  | 348 | 6 | - |  | 176 | 163 | 37 |
| *Albuminuria* | 51.72 | 43.1 | 5.18 |  | 45.4 | 43.17 | 11.43 |  | 92.72 | ** | ** |  | 98.86 | ** | - |  | 98.55 | 1.45 | - |  | 46.81 | 43.35 | 9.84 |
| *N* | 948 | 750 | 149 |  | 768 | 881 | 212 |  | 1695 | ** | ** |  | 1885 | ** | - |  | 1760 | 18 | - |  | 934 | 806 | 159 |
| *No Albuminuria* | 51.94 | 40.43 | 7.63 |  | 42.46 | 46.17 | 11.37 |  | 94.43 | ** | ** |  | 99.43 | ** | - |  | 98.77 | 1.23 | - |  | 49.18 | 42.44 | 8.37 |
| ***Non-Hispanic Blacks*** | | | | | | | | | | | | | | | | | | | | | | | |
|  | **rs2808630** | | |  | **rs1205** | | |  | **rs1800947*** | | |  | **rs3093058** | | |  | **rs3093066** | | |  | **rs1417938** | | |
| *N* | 159 | 86 | 8 |  | 172 | 71 | 8 |  | ** | ** | - |  | 189 | 61 | ** |  | 133 | 96 | 14 |  | 197 | 54 | ** |
| *Albuminuria* | 62.85 | 33.99 | 3.16 |  | 69.2 | 27.68 | 3.12 |  | 98 | ** | - |  | 69.75 | 27.65 | ** |  | 55.19 | 39.16 | 5.65 |  | 78.17 | 21.42 | ** |
| *N* | 972 | 333 | 26 |  | 822 | 412 | 80 |  | ** | ** | - |  | 938 | 366 | ** |  | 772 | 452 | 68 |  | 1048 | 280 | 22 |
| *No Albuminuria* | 73.03 | 25.02 | 1.95 |  | 62.41 | 31.52 | 6.07 |  | 99** | ** | - |  | 76.49 | 22.25 | ** |  | 60.77 | 34.08 | 5.15 |  | 77.62 | 20.74 | 1.63 |
| ***Mexicans Americans*** | | | | | | | | | | | | | | | | | | | | | | | |
|  | **rs2808630** | | |  | **rs1205** | | |  | **rs1800947*** | | |  | **rs3093058** | | |  | **rs3093066** | | |  | **rs1417938** | | |
| *N* | 155 | 85 | 10 |  | 97 | 127 | 29 |  | 235 | 7 | - |  | 243 | 8 | ** |  | 252 | ** | ** |  | 102 | 122 | 24 |
| *Albuminuria* | 60.19 | 34.39 | 5.32 |  | 42.82 | 46.59 | 10.58 |  | 97.53 | 2.47 | - |  | 95.6 | 4.11 | ** |  | 99.21 | ** | ** |  | 41.13 | 49.19 | 9.68 |
| *N* | 864 | 438 | 58 |  | 567 | 631 | 170 |  | 1284 | 54 | - |  | 1365 | 37 | ** |  | 1325 | ** | ** |  | 599 | 624 | 155 |
| *No Albuminuria* | 62.6 | 32.94 | 4.46 |  | 41.57 | 45.73 | 12.7 |  | 95.6 | 4.37 | - |  | 97.1 | 2.9 | ** |  | 96.61 | ** | ** |  | 43.49 | 45.23 | 11.27 |

** Data user agreement with the CDC does not allow outputs with counts less than 5.

***N:*** number of individuals with the specific genotype. ***GFP:*** genotype frequency percent.
